# Poor self-rated health in individuals with irritable bowel syndrome but no increased 10-year cardiovascular risk: results from a Swedish population-based screening program

**DOI:** 10.3389/fcvm.2026.1702012

**Published:** 2026-02-25

**Authors:** Emelie Stenman, Beata Borgström Bolmsjö, Clara Nilholm, Anton Grundberg, Kristina Sundquist

**Affiliations:** 1 Center for Primary Health Care Research, Department of Clinical Sciences Malmö, Lund University, Malmö, Sweden; 2 University Clinic Primary Care, Skåne University Hospital, Region Skåne, Skåne County, Sweden

**Keywords:** cardiovascular risk, lifestyle factors, middle-aged, SCORE2, self-rated health, targeted health dialogues

## Abstract

**Introduction:**

Irritable bowel syndrome (IBS) has been linked to poor health, high stress and diminished quality of life. Previous findings about associations between IBS and cardiovascular disease are, however, contradictory. Our objective was to examine 10-year cardiovascular risk, lifestyle factors, and self-rated health in people with IBS compared to a reference group.

**Methods:**

Sweden's southernmost county (Scania) has implemented targeted health dialogues (THDs) in primary health care for all 40- and 50-year-olds. Before the THD, participants completed a questionnaire about their health and lifestyle. Cholesterol, blood glucose, BMI and waist-hip-ratio were measured. Participants were also invited to a research project. The present study used baseline variables from 2021 to 2024 to examine the 10-year risk of cardiovascular events according to SCORE2, four health behaviors (diet, physical activity, smoking and alcohol), and self-rated health in THD-participants with and without IBS. Analyses took into account sex, level of education and place of birth.

**Results:**

The study population comprised 8 899 (56.5%) THD participants of which 592 (6.7%) had a diagnosis of IBS. Participants with IBS did not show an increased 10-year cardiovascular risk after adjusting for confounders. However, men with IBS had higher mean diastolic blood pressure (*p* = 0.030), and women with IBS had higher mean waist-hip-ratio compared to the reference group (*p* = 0.013). There were no overall differences shown in physical activity, smoking, or diet between participants with/without IBS, but women with IBS consumed less alcohol (*p* = 0.026). Poor self-rated health was significantly more common in both women and men with IBS (*p* < 0.001).

**Conclusion:**

IBS was not associated with an increased 10-year cardiovascular risk in this population, but further research is needed, preferably subtype-specific. THDs and similar interventions may provide opportunities to identify needs for healthcare support in people with IBS suffering from poor self-rated health.

## 1 Introduction

This cross-sectional study examines possible associations between irritable bowel syndrome (IBS) and 10-year cardiovascular risk, lifestyle factors, and self-rated health in 40- and 50-year-olds participating in a Swedish health screening and intervention program. IBS is a functional gastrointestinal disorder that is prevalent all over the world and causes considerable suffering for affected individuals. The prevalence differs depending on diagnostic criteria, populations and methods. However, a recent study, which covered all continents, found that most of the included countries had prevalences of IBS between 3%–5% according to the Rome IV diagnostic criteria (see definition below) (1).

The diagnosis of IBS is based on typical symptoms and the ruling out of organic diseases. Lack of biomarkers for the syndrome makes the diagnostics challenging (2). The first Rome criteria for functional gastrointestinal disorders, including IBS, were published in 1992 and have been updated subsequently. The current Rome IV criteria, established in 2016, require recurrent abdominal pain at least once weekly with two of the following: (1) relation to defecation, (2) change in stool frequency, or (3) change in the stool form (3). IBS-subtypes include IBS-C (constipation), IBS-D (diarrhea), IBS-M (mixed), and IBS-U (unclassified) (2, 3). IBS is more common in women and typically diagnosed before 50 years of age (4).

People with IBS are often limited by involuntary restrictions and problems, such as difficulties travelling or even leaving home, sleeping disturbances, pain, and a higher prevalence of comorbidities compared to control populations (4–6). These limitations could be stressful and potentially have a negative influence on long-term health, including cardiovascular outcomes. To our knowledge though, no evidence has so far been found for an association with cardiovascular or all-cause mortality (7–10). On the other hand, studies have shown associations between IBS and cardiovascular risk factors such as obesity (11) and metabolic syndrome (12–14). Studies have also suggested associations between IBS and high blood pressure (5, 12, 15), as well as genetic correlations with cardiovascular diseases (5). Thus, the findings regarding IBS and cardiovascular risk are paradoxical, and the relationships need to be further elucidated to identify possible needs of preventive efforts.

A broad implementation of so-called targeted health dialogues [THDs; health screening and intervention program (16)] in a Swedish county has provided an opportunity to study associations between IBS, cardiovascular risk factors, and lifestyle. The aim of the present study was to analyze cardiovascular risk as assessed by SCORE2, several other cardiovascular risk factors, including four health behaviors (dietary patterns, physical activity, smoking, and alcohol), and self-rated health in THD participants with IBS as compared to participants without the condition.

## 2 Materials and methods

### 2.1 Setting and research ethics

The study was performed in Scania, the southernmost county of Sweden, with about 1.4 million inhabitants and about 180 tax-financed primary healthcare centers (PHCCs). All inhabitants of Scania are registered at one PHCC, and it is mandatory for the PHCCs to participate in the THD program.

The study includes cross-sectional analyses from the baseline measurements in THDs performed in the period covering September 2021 through to 2024. All participants in the THDs were also invited to participate in the research project. Written informed consent was a prerequisite for taking part in the research project. The protocol was approved by the Swedish Ethical Review Authority (registration number 2020-02689 with later amendments) and registered at ClinicalTrials.gov, identifier: NCT04912739.

### 2.2 The THD method

The THD method was developed by two Swedish regions, Habo municipality and the West Bothnia province, already in the 1980s to prevent type 2 diabetes and cardiovascular disease (17, 18). The slightly modified method that is used in Scania has been described in detail previously (16). Each PHCC was encouraged to invite all their registered 40- and 50-year-olds to a THD. Those who wanted to participate filled in an electronic questionnaire about themselves regarding details such as place of birth, education, chronic diseases, heredity, health behaviors (tobacco, alcohol, diet, physical activity), and self-rated health. Two visits were booked to the PHCC. On the first visit, the participant took a fasting blood test to measure total cholesterol, low-density lipoprotein (LDL) cholesterol, high-density lipoprotein (HDL) cholesterol, and plasma glucose. Body mass index (BMI), waist-hip ratio, and sitting blood pressure (after five minutes rest) were also measured. The results from the questionnaire and measurements were automatically translated into a visual tool, a so-called health profile presented as a table with 13 risk-factor categories and four color-coded risk levels: green, yellow, orange, and red (16). Green indicates the lowest cardiovascular risk, while red represents the highest. On the second visit, the participants went through a one-hour THD led by a health dialogue coach, i.e., a registered nurse, dietician, occupational- or physiotherapist, or physician with a special education in the THD method. During the THD, lifestyle and cardiovascular risk factors were discussed, based on the participant's health profile, and solutions to possible health problems were suggested based on their life situation and preferences. When necessary, follow-up visits were booked or referrals were sent to appropriate specialists.

### 2.3 Participants and variables

For those who consented to take part in the research project, pseudonymized data from questionnaires and measurements were collected from a quality register within the County Council of Scania.

IBS diagnosis was defined by the International Classification of Diseases (ICD)-code K58 and collected from the medical records (from the year 2000 and onwards). IBS could also be self-reported from the question: “Has a doctor ascertained that you have or have had any other long-term illness?” with the follow-up response IBS. Self-reported IBS diagnoses were included because some participants may have received their diagnosis outside the Scania region prior to entering the regional healthcare system. In total, 10 participants (1.7% of IBS cases) reported an IBS diagnosis without a corresponding ICD code in the regional registers that were available to us. For the purposes of this study, ICD-coded IBS and self-reported IBS were treated equivalently in all analyses. Participants without an IBS diagnosis or self-reported IBS constituted the reference group.

Participants with a diagnosis of diabetes, International Classification of Diseases (ICD)-10 codes E10-E14 and/or self-reported diabetes, Crohn's disease (ICD-10 K50), ulcerative colitis (ICD-10 K51) or celiac disease (ICD-10 K90.0) were excluded since their risk factors and dietary patterns may be influenced by their condition [and the version of SCORE2 used is not intended for diabetes (19)].

The participants were also characterized by sex, age, level of education (≤9 years, upper secondary school, post-secondary school), and place of birth (Sweden, other European country, non-European country).

Fasting total, high-density lipoprotein (HDL), and low-density lipoprotein (LDL) cholesterol (mmol/L), fasting blood glucose (mmol/L), and systolic and diastolic blood pressure (mmHg) were expressed as mean values with standard deviations.

The prediction model SCORE2 was used to estimate the 10-year percentage risk of fatal and non-fatal cardiovascular events. SCORE2 combines the variables age, sex, smoking status, systolic blood pressure, and non-HDL cholesterol, and takes into account which European region the country belongs to (Sweden belongs to the moderate risk region) (19). The SCORE2 risk categories were defined in three levels: low risk = < 2.5% (40-year-olds), < 5% (50-year-olds); medium risk = 2.5 to < 7.5% (40-year-olds), 5 to < 10% (50-year-olds); and high risk = ≥ 7.5% (40-year-olds), ≥ 10% (50-year-olds).

Overweight was defined as BMI ≥ 25 kg/m^2^ and obesity as BMI ≥ 30 kg/m^2^ (20).

High waist–hip ratio was defined as ≥ 0.85 for women and ≥ 0.90 for men according to the WHO's (2008) definition (21).

The diet part of the questionnaire was an adapted version of a previously validated questionnaire (22), and the diet score in the health profile was calculated from a fat- and fiber index based on questions concerning food intake. In addition, if the participants answered that they were having “sweets, chocolate, or sugar-sweetened drinks” ≥ 2 times per day or “cakes or cookies” ≥ 2 times per day, or both categories one time per day, they were moved one step to the right in the health profile, thus towards an increased cardiovascular risk. To further analyze the dietary patterns in participants with or without an IBS diagnosis, we also compared each question in the diet part of the questionnaire separately. This also included the question “Do you eat any special diet for medical or other reasons?”. If the answer was yes, the participant was asked to describe the diet in the free text box provided.

Smoking was defined as daily cigarette smoking.

Physical activity was assessed by the question: *How physically active are you in your leisure time?* It had four response alternatives: (1) sedentary leisure time, (2) moderate exercise, (3) strenuous exercise, and (4) hard exercise. Participants who chose alternatives 2 or 3 were asked to respond to follow-up questions during the health dialogue regarding the mode of transport to work and other leisure time physical activities. Physical activity was reported as minutes per week and season, multiplied by specific energy factors per activity and reported as kilocalories per week (kcal/week) (23). Burning < 2000 kcal/week in leisure time was deemed to be insufficient physical activity according to the health profile.

Excessive alcohol intake was defined as drinking ≥ 5 (women) or ≥ 7 (men) standard glasses of alcohol (equivalent to 12–15 cl wine) per week. Drinking ≥ 4 (women) or ≥ 5 (men) standard glasses per occasion at least monthly was also categorized as excessive alcohol intake.

Self-rated health was estimated by a single question asking *How do you assess your general health condition?* with the response alternatives: (1) very good, (2) good, (3) fair, (4) poor and (5) very poor. The responses to single questions regarding self-rated health of this type have previously been shown to be associated with mortality risk (24).

### 2.4 Statistical analysis

Participant characteristics are presented with numbers and percentages for categorical variables and with means and standard deviations (SD), or medians with inter-quartile ranges (IQR) for continuous variables. Differences in characteristics between participants with or without IBS were tested using Chi-squared tests for categorical variables and Welch's *t*-tests for continuous variables.

In order to adjust for sex, level of education and place of origin, we performed additional analyses using linear regression, or logistic regression where odds ratios (OR) with 95% confidence intervals (CI) were calculated in three steps: model 1: unadjusted, model 2: adjusted for sex, and model 3: adjusted for sex, level of education and place of origin. The SCORE2 values were natural log transformed. Self-rated health was dichotomized into “very good/good” vs. “fair/poor/very poor”. We chose not to adjust for age, as this would have resulted in groups that were too small. Moreover, no significant difference in IBS prevalence was observed between the age groups. All analyses were performed as complete-case analyses and missing values were not imputed. The chosen level of statistical significance was 0.05. All statistical analyses were done in R version 4.4.2 (R Core Team, 2024).

## 3 Results

**Figure 1:**
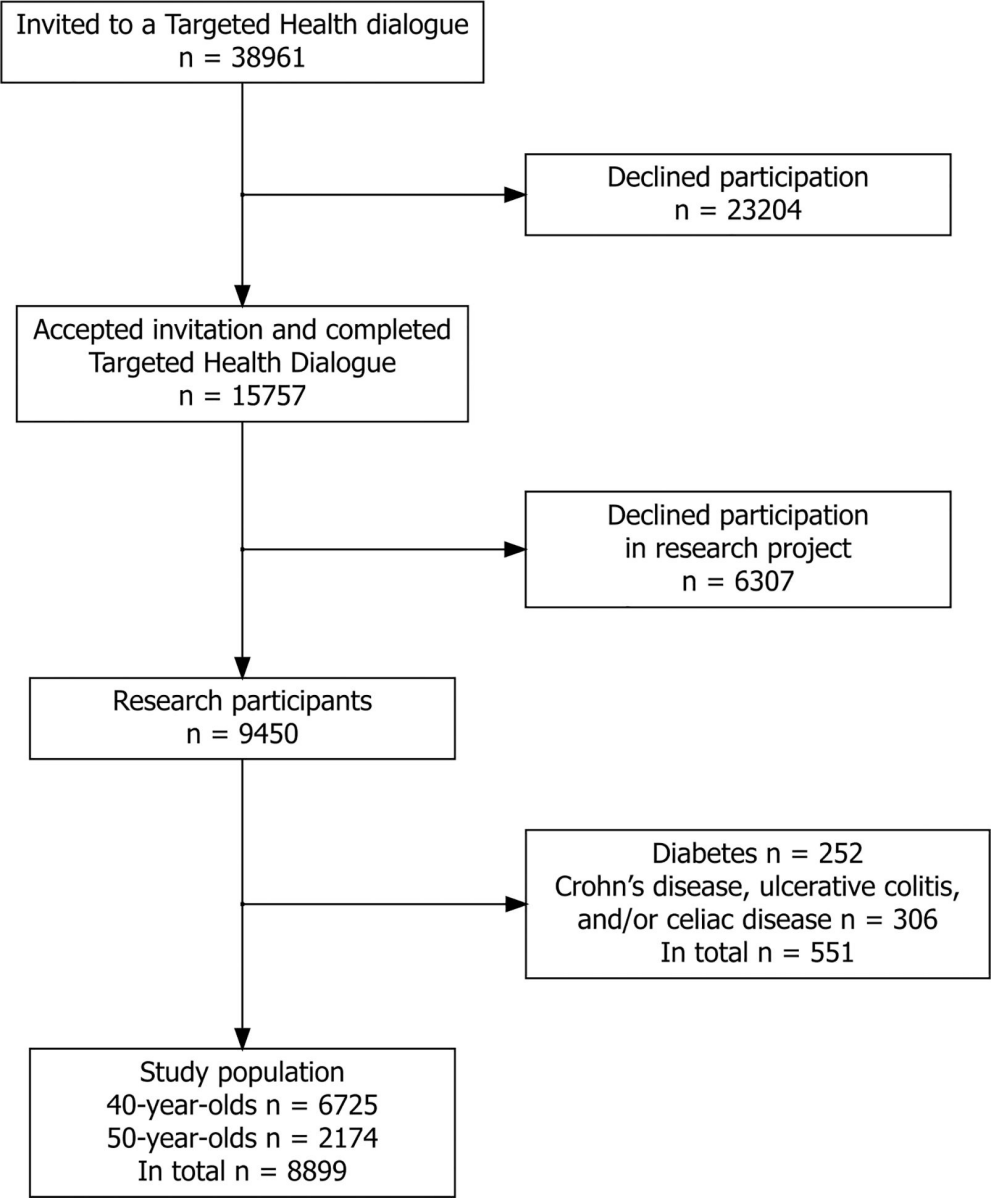
Flow chart showing the recruitment of study participants.

A total of 38,961 40- and 50-year-olds were invited to a THD, and 15,757 (40.4%) of them completed a THD during the study period. Of those who completed a THD, 9,450 (60.0%) consented to take part in the research project. After exclusion of 551 participants due to diagnosis of diabetes and/or Crohn's disease, ulcerative colitis, and/or celiac disease, the final study population was comprised of 8,899 participants, of which 75.6% were 40 years old (Figure 1, Table 1).

**Table 1:** Baseline characteristics of the study population and cardiovascular risk factors in participants with and without IBS.

Characteristics	All			Men			Women		
	No IBS *N* = 8,307	IBS *N* = 592	*p*-value	No IBS *N* = 3,786	IBS *N* = 191	*p*-value	No IBS *N* = 4,521	IBS *N* = 401	*p*-value
Sex, *n* (%)			**<0**.**001**						
Men	3,786 (45.6)	191 (32.3)							
Women	4,521 (54.4)	401 (67.7)							
Age group, *n* (%)			0.148			0.363			0.242
40	6,263 (75.4)	462 (78.0)		2,864 (75.6)	150 (78.5)		3,399 (75.2)	312 (77.8)	
50	2,044 (24.6)	130 (22.0)		922 (24.4)	41 (21.5)		1,122 (24.8)	89 (22.2)	
Level of education, *n* (%)			0.637			0.198			**0**.**023**
≤9 years	464 (5.6)	38 (6.4)		248 (6.6)	8 (4.2)		216 (4.8)	30 (7.5)	
Upper secondary school	2,432 (29.3)	167 (28.2)		1,357 (35.8)	62 (32.5)		1,075 (23.8)	105 (26.2)	
Post-secondary school	5,390 (64.9)	386 (65.2)		2,176 (57.5)	121 (63.4)		3,214 (71.1)	265 (66.1)	
Missing	21 (0.3)	1 (0.2)		5 (0.1)	0 (0.0)		16 (0.4)	1 (0.2)	
Place of origin, *n* (%)			**<0**.**001**			0.070			**0**.**001**
Sweden	5,749 (69.2)	455 (76.9)		2,636 (69.6)	143 (74.9)		3,113 (68.9)	312 (77.8)	
Other European country	1,090 (13.1)	51 (8.6)		492 (13.0)	14 (7.3)		598 (13.2)	37 (9.2)	
Non-European country	1,447 (17.4)	84 (14.2)		652 (17.2)	33 (17.3)		795 (17.6)	51 (12.7)	
Missing	21 (0.3)	2 (0.3)		6 (0.2)	1 (0.5)		15 (0.3)	1 (0.2)	
Total cholesterol, mmol/L, mean (SD)	4.82 (0.88)	4.72 (0.92)	**0**.**013**	4.97 (0.93)	4.96 (0.98)	0.876	4.69 (0.82)	4.61 (0.87)	0.066
HDL cholesterol, mmol/L, mean (SD)	1.45 (0.49)	1.44 (0.43)	0.981	1.30 (0.50)	1.27 (0.41)	0.297	1.56 (0.45)	1.53 (0.41)	0.097
LDL cholesterol, mmol/L, mean (SD)	3.29 (0.92)	3.18 (0.89)	**0**.**004**	3.54 (0.94)	3.54 (0.87)	0.991	3.08 (0.85)	3.01 (0.85)	0.093
fP-Glucose, mmol/L, mean (SD)	5.39 (0.51)	5.39 (0.57)	0.919	5.49 (0.54)	5.54 (0.67)	0.295	5.31 (0.47)	5.31 (0.51)	0.766
Systolic blood pressure, mmHg, mean (SD)	123.61 (14.80)	122.37 (15.24)	0.058	128.99 (14.23)	128.83 (13.99)	0.881	119.11 (13.71)	119.26 (14.86)	0.837
Diastolic blood pressure, mmHg, mean (SD)	79.80 (10.41)	79.77 (10.91)	0.948	81.95 (10.30)	83.64 (10.45)	**0**.**030**	78.00 (10.16)	77.90 (10.65)	0.861
SCORE2, median (IQR)	1.33 (1.51)	1.05 (1.28)	**<0**.**001**	2.14 (1.65)	2.11 (1.59)	0.521	0.73 (0.69)	0.73 (0.57)	0.945
SCORE2^a^, *n* (%)			0.349			0.570			0.467
Low risk	7,353 (88.5)	530 (89.5)		2,915 (77.0)	142 (74.3)		4,438 (98.2)	388 (96.8)	
Medium risk	868 (10.4)	51 (8.6)		816 (21.6)	45 (23.6)		52 (1.2)	6 (1.5)	
High risk	18 (0.2)	1 (0.2)		18 (0.5)	1 (0.5)		0 (0.0)	0 (0.0)	
Missing	68 (0.8)	10 (1.7)		37 (1.0)	3 (1.6)		31 (0.7)	7 (1.7)	

Bold *p*-values denote statistical significance.

^a^ SCORE2 risk categories are defined as: Low risk = <2.5% (40-year-olds), <5% (50-year-olds). Medium risk = 2.5 to <7.5% (40-year-olds), 5 to <10% (50-year-olds). High risk = ≥7.5% (40-year-olds), ≥10% (50-year-olds).

A total of 592 participants (6.7%) had a diagnosis of IBS, with a slightly higher proportion of IBS among 40-year-olds (6.9% vs. 6.0% in 50-year-olds). IBS was significantly more common among women than men (*p* < 0.001), and among participants born in Sweden compared to foreign-born (*p* < 0.001). Regarding educational level, women with IBS tended to have a lower education level compared to women without IBS (*p* = 0.023). This was not seen in men, where the trend for educational level was rather the opposite, with higher education level among those with IBS, although this was non-significant (Table 1).

When analyzing crude data, participants with IBS had significantly lower mean total cholesterol (*p* = 0.013), mean LDL cholesterol (*p* = 0.004) and median SCORE2 level (*p* < 0.001) compared to those without IBS. The significances disappeared, however, when adjusted for sex, level of education and place of birth (data not shown). Men with IBS had a slightly higher mean diastolic blood pressure compared to men without IBS (*p* = 0.030) (Table 1). There was no difference in BMI between those with IBS and those without IBS, but the mean waist-hip ratio was significantly higher among women with IBS compared to women without IBS (*p* = 0.013) (Table 2).

**Table 2:** Anthropometrics, health behaviors and self-rated health in participants with and without IBS.

Characteristics	All			Men			Women		
	No IBS *N* = 8,307	IBS *N* = 592	*p*-value	No IBS *N* = 3,786	IBS *N* = 191	*p*-value	No IBS *N* = 4,521	IBS *N* = 401	*p*-value
BMI, *n* (%)			0.529			0.829			0.918
<25 kg/m^2^	3,603 (43.4)	265 (44.8)		1,307 (34.5)	66 (34.6)		2,296 (50.8)	199 (49.6)	
25–29.9 kg/m^2^	3,001 (36.1)	200 (33.8)		1,685 (44.5)	82 (42.9)		1,316 (29.1)	118 (29.4)	
≥30 kg/m^2^	1,672 (20.1)	124 (20.9)		785 (20.7)	43 (22.5)		887 (19.6)	81 (20.2)	
Missing	31 (0.4)	3 (0.5)		9 (0.2)	0 (0.0)		22 (0.5)	3 (0.7)	
Waist-hip ratio, mean (SD)	0.86 (0.09)	0.86 (0.10)	0.617	0.91 (0.08)	0.92 (0.08)	0.209	0.81 (0.08)	0.83 (0.10)	0.013
Diet (health profile), *n* (%)			0.689			0.275			0.212
1 (green)	2,568 (30.9)	188 (31.8)		1,191 (31.5)	66 (34.6)		1,377 (30.5)	122 (30.4)	
2 (yellow)	2,244 (27.0)	149 (25.2)		970 (25.6)	52 (27.2)		1,274 (28.2)	97 (24.2)	
3 (orange)	2,183 (26.3)	154 (26.0)		1,052 (27.8)	43 (22.5)		1,131 (25.0)	111 (27.7)	
Missing	1,312 (15.8)	101 (17.1)		573 (15.1)	30 (15.7)		739 (16.3)	71 (17.7)	
Daily cigarette smoking, *n* (%)	600 (7.2)	35 (5.9)	0.231	304 (8.0)	10 (5.2)	0.162	296 (6.5)	25 (6.2)	0.808
Physical activity, *n* (%)			0.142			0.808			0.106
Sufficient	2,726 (32.8)	175 (29.6)		1,238 (32.7)	61 (31.9)		1,488 (32.9)	114 (28.4)	
Insufficient	5,362 (64.5)	395 (66.7)		2,440 (64.4)	125 (65.4)		2,922 (64.6)	270 (67.3)	
Missing	219 (2.6)	22 (3.7)		108 (2.9)	5 (2.6)		111 (2.5)	17 (4.2)	
Alcohol consumption, *n* (%)			0.030			0.854			0.026
Normal	6,420 (77.3)	472 (79.7)		2,706 (71.5)	139 (72.8)		3,714 (82.1)	333 (83.0)	
Excessive	1,456 (17.5)	82 (13.9)		829 (21.9)	44 (23.0)		627 (13.9)	38 (9.5)	
Missing	431 (5.2)	38 (6.4)		251 (6.6)	8 (4.2)		180 (4.0)	30 (7.5)	
Do you eat any special diet for medical or other reasons? (Select only one option)			<0.001			0.003			<0.001
Yes	849 (10.2)	129 (21.8)		260 (6.9)	24 (12.6)		589 (13.0)	105 (26.2)	
No	7,443 (89.6)	462 (78.0)		3,520 (93.0)	167 (87.4)		3,923 (86.8)	295 (73.6)	
Missing	15 (0.2)	1 (0.2)		6 (0.2)	0 (0.0)		9 (0.2)	1 (0.2)	
How do you assess your general health condition? *n* (%)			<0.001			<0.001			<0.001
Very good	1,347 (16.2)	50 (8.4)		628 (16.6)	16 (8.4)		719 (15.9)	34 (8.5)	
Good	4,868 (58.6)	301 (50.8)		2,265 (59.8)	105 (55.0)		2,603 (57.6)	196 (48.9)	
Fair	1,783 (21.5)	184 (31.1)		777 (20.5)	56 (29.3)		1,006 (22.3)	128 (31.9)	
Poor	262 (3.2)	49 (8.3)		95 (2.5)	11 (5.8)		167 (3.7)	38 (9.5)	
Very poor	30 (0.4)	5 (0.8)		16 (0.4)	1 (0.5)		14 (0.3)	4 (1.0)	
Missing	17 (0.2)	3 (0.5)		5 (0.1)	2 (1.0)		12 (0.3)	1 (0.2)	

Bold *p*-values denote statistical significance.

Regarding health behaviors according to the health profile, there were no overall differences in physical activity or smoking between those with IBS and those without. There were fewer participants with excessive alcohol consumption among participants with IBS (*p* = 0.030). The difference remained significant after adjusting for sex, level of education and place of birth (data not shown) and was driven by women with IBS who had a more moderate alcohol consumption compared to the reference group of women (*p* = 0.026) (Table 2).

**Figure 2:**
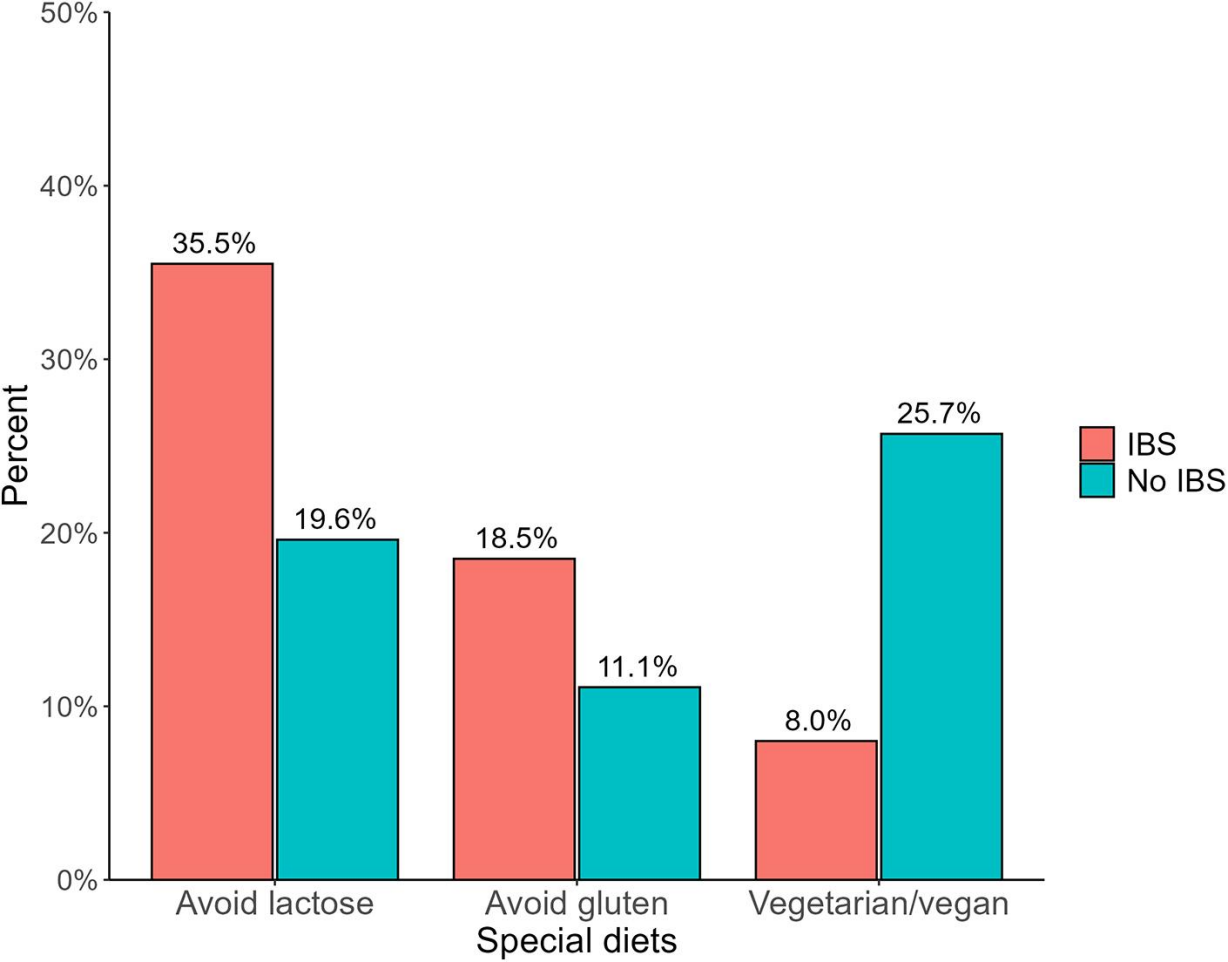
Self-reported special diets in participants with and without IBS.

Regarding diet, the cardiovascular risk level in the health profile did not differ between those with IBS and the reference group. However, both women (*p* < 0.001) and men (*p* = 0.003) with IBS answered that they followed a special diet for medical or other reasons more often than participants without IBS (Table 2). Among participants with IBS who followed a special diet, 35.5% reported that they avoided lactose, and 18.5% avoided gluten. 8.0% were vegetarians or vegans. The corresponding figures among participants without IBS who followed a special diet were 19.6%, 11.1%, and 25.7% respectively (Figure 2).

When analyzing the food questionnaire in detail, a few differences stood out. The only significant differences between those with IBS and those in the reference group were the following: (1) More participants with IBS did not eat bread at all (9.3% vs. 5.8% in the reference group) and those with IBS more often ate whole grain bread or crisp bread and less often loaf/farmhouse bread compared to the reference group (*p* < 0.001), (2) Participants with IBS reported eating chocolate slightly more often than those without IBS (*p* = 0.028), which was driven by women with IBS (*p* = 0.011), (3) Participants with IBS reported cutting off visible fat when eating meat more often compared to the reference group (*p* = 0.007). Apart from these variables, we could not detect any differences in eating habits with the THD food questionnaire (Supplementary Table S1).

Both men and women with IBS had significantly poorer self-rated health (*p* < 0.001), with only 59.2% reporting good or very good health compared to 74.8% among those without IBS (Table 2). After adjusting for sex, education level, and country of origin, the odds of having poor general health were still double for those with IBS [OR = 2.03 (CI: 1.70–2.41); *p* < 0.001; data not shown].

## 4 Discussion

In this population-based study of 40- and 50-year-olds, no increased 10-year cardiovascular risk was observed in participants with IBS, as assessed by SCORE2. However, women with IBS had a higher mean waist-hip ratio, and men with IBS had a higher mean diastolic blood pressure compared to the reference group. Regarding health behaviors, women with IBS consumed less alcohol on a group level. Participants with IBS more often reported that they followed a special diet, which could be discerned in the form of small differences in bread, chocolate, and visible fat consumption. They also more often reported avoiding lactose and/or gluten. Finally, our results confirm previous findings that both women and men with IBS suffer from significantly poorer self-rated health than those without the disorder.

The similar SCORE2 levels in participants with IBS and the reference group are supported by previous studies that showed no increased cardiovascular mortality in patients with IBS (8, 9). There are, however, some uncertainties regarding this relationship. In the nationwide Swedish cohort study by Staller et al. (8), a higher percentage of individuals with IBS had cardiovascular disease at baseline compared to the reference group. Cardiovascular mortality was also slightly higher in the IBS group before adjusting for comorbidity including cardiovascular disease (8) (a methodological step that could be regarded as reasonable, but also one that may obscure a potential pathway between IBS and cardiovascular mortality). The other previous study of IBS and cardiovascular mortality failed to detect an increased risk of cardiovascular mortality in individuals with IBS, but they identified a potential common biomarker for IBS-D, glycoprotein acetyls (GlycA). GlycA has also been shown to be related to cardiovascular mortality and the subgroup of IBS patients with elevated serum GlycA had a higher mortality rate (9). Thus, there may be a discrepancy in cardiovascular risk depending on the type of IBS. Unfortunately, we did not have access to reliable information about IBS subtypes in our study population, a limitation that should be considered when interpreting the findings. Future research applying SCORE2 in the context of IBS should aim to incorporate validated subtype information and, if feasible, complementary biomarkers and/or risk modifiers that can improve model performance. Such approaches may help identify non-conventional or inflammation-related pathways that contribute to cardiovascular risk among individuals with different subtypes of IBS.

Women with IBS in our study had similar proportions of overweight and obesity as the reference population, but they had a slightly higher average waist-hip-ratio. IBS has been related to abdominal obesity before (11, 12, 14, 25), and since a high waist-hip-ratio is a known predictor of cardiovascular disease, especially in middle-aged women (26), our findings may be of long-term importance from a cardiovascular perspective. However, an alternative explanation for the higher waist-hip-ratio seen in our study is abdominal bloating, which is a common symptom in IBS (4). Yet another finding related to cardiovascular risk was that men with IBS had a slight, but significantly, higher mean diastolic blood pressure compared to the reference group, which may reflect a higher stress level in men with IBS (4, 27). Diastolic hypertension is a risk factor for cardiovascular events and mortality, especially in younger people (28). These findings support a need for further research on cardiovascular risk factors related to IBS, if possible, taking subtypes into account.

In the comparison of lifestyle factors, we found that women with IBS were significantly less often heavy drinkers compared to the reference group. Men with IBS were less often cigarette smokers compared to the reference group, although not to a significant degree. There is, to our knowledge, no conclusive research about the effects of alcohol or smoking on IBS symptoms, although a few studies have suggested an aggravation of abdominal symptoms by these behaviors (29), which may explain our results. The health profile diet score did not differ for participants with IBS. Nevertheless, they more often reported following a special diet with a large part avoiding lactose and/or gluten. This is in accordance with a meta-analysis showing that the overall intake of macro- and micronutrients in patients with IBS followed dietary recommendations, with the exception of suboptimal intake of fiber and vitamin D (30), which hypothetically may be connected to lower intake of gluten and vitamin D-fortified dairy products.

It is reassuring that current research has not yet identified any negative impact of IBS on mortality (7–9). Nevertheless, poor self-rated health, which was commonly reported among both women and men with IBS in our study, can significantly affect daily life and well-being. Although the THDs mainly focus on cardiovascular risk, they can also be used for discussions regarding other health problems and the participants are referred to appropriate specialists when needed. In IBS, this can be an opportunity to achieve contact with e.g., dieticians or physicians that may be able to provide relief, by dietary advice or physical examinations that may rule out serious diseases, which is a common worry in IBS patients, such as fear of cancer (31).

### 4.1 Limitations

The study population was derived from data involving participants in a THD program, which aims to include all 40- and 50-year-old inhabitants living in the county of Scania, southern Sweden. There is, however, a small but significant under-representation of men, low-educated, and foreign-born participants in the THDs for unknown reasons (16). This may influence the external validity if, for instance, the THD participants were more health-conscious compared to the non-participants. The cardiovascular risk profile may differ in studies of IBS in e.g., socioeconomically disadvantaged populations or in subgroups with severe IBS cases only. On the other hand, no active selection was made on e.g., diagnoses or any other variable.

Clinical diagnoses were only available from the year 2000 onwards; thus, we may have missed some IBS diagnoses that were registered more than 20 years earlier. Hopefully, most of these were identified in the questionnaire. IBS is also a disease with diffuse symptoms; thus, there may be some individuals in the reference group that were not yet diagnosed. There may also be individuals classified as having IBS that in fact have alternative conditions, such as other functional gastrointestinal disorders (e.g., functional diarrhea, functional constipation), undetected inflammatory bowel disease, celiac disease, or gastrointestinal symptoms driven by non-somatic factors. Given the heterogeneous nature of IBS, some degree of overlap between the groups is expected, which could attenuate potential differences in the associations between IBS and cardiovascular disease risk. Nevertheless, the progressively refined Rome criteria likely reduce this risk to some degree (2).

Health behaviors were self-reported, which implies a risk of self-report bias. In addition, a slight risk of differential self-reporting between IBS and non-IBS participants cannot be excluded. The other measurements, including anthropometry, were, however objectively measured, which strengthens the validity of the cardiovascular risk assessment. Another limitation related to self-reporting is that factors such as depression, stress, anxiety, and central sensitization may contribute to the high levels of poor self-rated health observed in individuals with IBS. Future research should aim to clarify the underlying mechanisms linking IBS to poor self-rated health, which may help identify potential targets for intervention and improvement.

The questionnaire used in the THDs was not designed for IBS specifically, but for detecting cardiovascular risk behavior. Detailed dietary patterns, such as intake of fermentable oligosaccharides, disaccharides, monosaccharides and polyols (FODMAP), were not assessed. With a diet questionnaire targeting IBS, more differences in dietary patterns may have been found.

The most important limitation of our study is its cross-sectional design, which restricts the causal conclusions that can be drawn. Although SCORE2 is a validated tool for estimating 10-year cardiovascular risk (19), only a longitudinal design can determine the actual cumulative cardiovascular risk and incidence associated with chronic IBS. Similarly, for self-rated health, causal pathways cannot be established in a cross-sectional context. At the same time, longitudinal studies in this field face challenges of their own. IBS symptoms often develop gradually and may be present for many years before a formal diagnosis is made. This complicates the temporal ordering of symptom onset, diagnosis, and subsequent outcomes, and may introduce misclassification or reverse-causation issues. Thus, while longitudinal approaches are needed to strengthen causal inference, they are not without methodological difficulties in the context of IBS.

Finally, we lacked reliable data on IBS subtype. Hopefully, subtypes will be more commonly available in the clinical registers in the future, after the introduction of the later Rome diagnostic criteria, which are more specific in this regard (2). Despite these limitations, the study also has many strengths, such as the population-based nature of the study sample of both women and men, and the inclusion of several cardiovascular risk factors, as well as the accounting for potential confounding.

### 4.2 Conclusion

The present study found no evidence of increased 10-year cardiovascular risk, as estimated by SCORE2, among 40- and 50-year-olds with IBS compared to the reference group. This is in accordance with previous research that suggests no increased cardiovascular mortality in people with IBS. However, positive associations were observed between IBS and certain individual cardiovascular risk factors, indicating a need for further research, particularly across different IBS subtypes.

Poor self-rated health was significantly more prevalent among both women and men with IBS, underscoring the negative impact of IBS symptoms on perceived health. Primary care screening programs, such as the THDs, may play a valuable role in identifying health challenges in individuals with IBS and guiding supportive interventions to enhance well-being. The broader implications of such programs for improving patient outcomes merit continued exploration.

## Data Availability

The datasets presented in this article are not readily available because of research ethics regulations. Requests to access the datasets should be directed to the corresponding author.
