# Supplementary material for: Poor self-rated health in individuals with irritable bowel syndrome but no increased 10-year cardiovascular risk: results from a Swedish population-based screening program
Source: Front Cardiovasc Med. 2026 Feb 25;13:1702012. doi: 10.3389/fcvm.2026.1702012 (PMC12976889; doi:10.3389/fcvm.2026.1702012)
Supplement: Supplementary file 1 [file Datasheet1.pdf]

Supplementary Table 1. Food questionnaire used in the targeted health dialogues (freely translated from Swedish to English, partly using an AI-tool).

	All			Men			Women		
**Characteristics**	**No IBS N=8307**	**IBS N=592**	**p-value**	**No IBS N=3786**	**IBS N=191**	**p-value**	**No IBS N=4521**	**IBS N=401**	**p-value**
I select Keyhole*-labelled options when buying bread/cereals/grains, meat and charcuterie products, dairy products, and fats. (Select only one option)			0.995			0.903			0.393
Yes	1151 (13.9)	82 (13.9)		382 (10.1)	21 (11.0)		769 (17.0)	61 (15.2)	
Partially/Sometimes	4790 (57.7)	342 (57.8)		2068 (54.6)	104 (54.5)		2722 (60.2)	238 (59.4)	
No	2348 (28.3)	166 (28.0)		1328 (35.1)	65 (34.0)		1020 (22.6)	101 (25.2)	
Missing	18 (0.2)	2 (0.3)		8 (0.2)	1 (0.5)		10 (0.2)	1 (0.2)	
What type of fat do you most often use in cooking? (By oil, we mean, for example, olive oil or rapeseed oil) (Select only one option)			0.286			0.641			0.222
Oil	4890 (58.9)	326 (55.1)		2151 (56.8)	100 (52.4)		2739 (60.6)	226 (56.4)	
Liquid cooking fat (not oil)	1556 (18.7)	125 (21.1)		720 (19.0)	39 (20.4)		836 (18.5)	86 (21.4)	
Solid margarine	217 (2.6)	22 (3.7)		102 (2.7)	5 (2.6)		115 (2.5)	17 (4.2)	
Butter	1354 (16.3)	97 (16.4)		660 (17.4)	36 (18.8)		694 (15.4)	61 (15.2)	
Coconut fat	73 (0.9)	3 (0.5)		27 (0.7)	1 (0.5)		46 (1.0)	2 (0.5)	
Don't know	77 (0.9)	7 (1.2)		62 (1.6)	5 (2.6)		15 (0.3)	2 (0.5)	
Other cooking fat	127 (1.5)	11 (1.9)		58 (1.5)	5 (2.6)		69 (1.5)	6 (1.5)	
Missing	13 (0.2)	1 (0.2)		6 (0.2)	0 (0.0)		7 (0.2)	1 (0.2)	
How often do you eat nuts or seeds? (Select only one option)			0.728			0.801			0.939
Daily	1039 (12.5)	83 (14.0)		381 (10.1)	22 (11.5)		658 (14.6)	61 (15.2)	
A few times a week	2977 (35.8)	204 (34.5)		1261 (33.3)	58 (30.4)		1716 (38.0)	146 (36.4)	
Once a week or less often	3792 (45.6)	269 (45.4)		1890 (49.9)	99 (51.8)		1902 (42.1)	170 (42.4)	
Never	482 (5.8)	34 (5.7)		247 (6.5)	12 (6.3)		235 (5.2)	22 (5.5)	
Missing	17 (0.2)	2 (0.3)		7 (0.2)	0 (0.0)		10 (0.2)	2 (0.5)	
How often do you eat fast food, such as pizza and hamburgers? (Select only one option)			0.561			0.248			0.152

Twice a day or more often	14 (0.2)	1 (0.2)		10 (0.3)	0 (0.0)		4 (0.1)	1 (0.2)	
Once a day	73 (0.9)	6 (1.0)		54 (1.4)	2 (1.0)		19 (0.4)	4 (1.0)	
A few times a week	844 (10.2)	63 (10.6)		628 (16.6)	38 (19.9)		216 (4.8)	25 (6.2)	
Once a week or less often	6929 (83.4)	482 (81.4)		2966 (78.3)	140 (73.3)		3963 (87.7)	342 (85.3)	
Never	437 (5.3)	39 (6.6)		124 (3.3)	11 (5.8)		313 (6.9)	28 (7.0)	
Missing	10 (0.1)	1 (0.2)		4 (0.1)	0 (0.0)		6 (0.1)	1 (0.2)	
I mostly eat (Select only one option)			**<0.001**			0.052			**0.034**
White bread	1626 (19.6)	108 (18.2)		957 (25.3)	42 (22.0)		669 (14.8)	66 (16.5)	
Loaf, farmhouse bread	2140 (25.8)	117 (19.8)		1168 (30.9)	48 (25.1)		972 (21.5)	69 (17.2)	
Coarse rye bread	1234 (14.9)	85 (14.4)		549 (14.5)	32 (16.8)		685 (15.2)	53 (13.2)	
Whole grain, crispbread	2807 (33.8)	225 (38.0)		900 (23.8)	51 (26.7)		1907 (42.2)	174 (43.4)	
I don't eat bread	481 (5.8)	55 (9.3)		203 (5.4)	18 (9.4)		278 (6.1)	37 (9.2)	
Missing	19 (0.2)	2 (0.3)		9 (0.2)	0 (0.0)		10 (0.2)	2 (0.5)	
On my sandwiches, I usually spread (Select only one option)			0.887			0.653			0.729
Butter, Bregott**, or margarine	5473 (65.9)	382 (64.5)		2455 (64.8)	121 (63.4)		3018 (66.8)	261 (65.1)	
Light margarine (maximum 40% fat, e.g., Becel, Lätta, Lätt&Lagom)	1576 (19.0)	115 (19.4)		750 (19.8)	36 (18.8)		826 (18.3)	79 (19.7)	
No fat	1221 (14.7)	89 (15.0)		563 (14.9)	33 (17.3)		658 (14.6)	56 (14.0)	
Missing	37 (0.4)	6 (1.0)		18 (0.5)	1 (0.5)		19 (0.4)	5 (1.2)	
On a sandwich, I usually spread (Select only one option)			0.097			0.412			0.765
At least the equivalent of one portion pack (10 g)	2241 (27.0)	136 (23.0)		1233 (32.6)	52 (27.2)		1008 (22.3)	84 (20.9)	
About half a portion pack of fat	3131 (37.7)	224 (37.8)		1373 (36.3)	71 (37.2)		1758 (38.9)	153 (38.2)	
Less than half a portion pack	1623 (19.5)	135 (22.8)		578 (15.3)	34 (17.8)		1045 (23.1)	101 (25.2)	
No fat	1266 (15.2)	91 (15.4)		579 (15.3)	33 (17.3)		687 (15.2)	58 (14.5)	
Missing	46 (0.6)	6 (1.0)		23 (0.6)	1 (0.5)		23 (0.5)	5 (1.2)	
For sandwich toppings, I mostly have (Select only one option)			0.423			0.616			0.667
Cheese (>17% fat content), sausage, liver pâté	3509 (42.2)	248 (41.9)		1689 (44.6)	84 (44.0)		1820 (40.3)	164 (40.9)	

Lean or smoked ham, low-fat cheese (≤17% fat content), soft cheese, hamburger meat, mackerel, whey cheese	1481 (17.8)	104 (17.6)		637 (16.8)	32 (16.8)		844 (18.7)	72 (18.0)	
Use the above two options equally often	2076 (25.0)	145 (24.5)		992 (26.2)	51 (26.7)		1084 (24.0)	94 (23.4)	
Fruit or vegetable toppings	601 (7.2)	36 (6.1)		193 (5.1)	6 (3.1)		408 (9.0)	30 (7.5)	
No toppings	599 (7.2)	54 (9.1)		254 (6.7)	17 (8.9)		345 (7.6)	37 (9.2)	
Missing	41 (0.5)	5 (0.8)		21 (0.6)	1 (0.5)		20 (0.4)	4 (1.0)	
If I eat a cheese sandwich, I usually put on (Select only one option)			0.489			0.796			0.943
At least 3 slices of cheese (Note: equivalent to the portion slices you get at, for example, a restaurant)	461 (5.5)	26 (4.4)		303 (8.0)	13 (6.8)		158 (3.5)	13 (3.2)	
2 slices of cheese	4130 (49.7)	289 (48.8)		1999 (52.8)	99 (51.8)		2131 (47.1)	190 (47.4)	
0–1 slice of cheese	2870 (34.5)	208 (35.1)		1121 (29.6)	57 (29.8)		1749 (38.7)	151 (37.7)	
I don't eat cheese sandwiches	819 (9.9)	66 (11.1)		347 (9.2)	21 (11.0)		472 (10.4)	45 (11.2)	
Missing	27 (0.3)	3 (0.5)		16 (0.4)	1 (0.5)		11 (0.2)	2 (0.5)	
Of bread (including crispbread), I eat every day (Select only one option)			0.505			0.384			0.756
0-2 slices of bread	6385 (76.9)	469 (79.2)		2597 (68.6)	140 (73.3)		3788 (83.8)	329 (82.0)	
3-5 slices of bread	1759 (21.2)	111 (18.8)		1068 (28.2)	44 (23.0)		691 (15.3)	67 (16.7)	
6-9 slices of bread	117 (1.4)	7 (1.2)		97 (2.6)	6 (3.1)		20 (0.4)	1 (0.2)	
At least 10 slices of bread	13 (0.2)	0 (0.0)		10 (0.3)	0 (0.0)		3 (0.1)	0 (0.0)	
Missing	33 (0.4)	5 (0.8)		14 (0.4)	1 (0.5)		19 (0.4)	4 (1.0)	
Of milk, sour milk, yoghurt, I drink/eat (Select only one option)			0.181			0.627			0.624
More than 1 litre daily	112 (1.3)	4 (0.7)		85 (2.2)	2 (1.0)		27 (0.6)	2 (0.5)	
3–10 dl daily	1972 (23.7)	126 (21.3)		1101 (29.1)	51 (26.7)		871 (19.3)	75 (18.7)	
At most 2 dl daily	3577 (43.1)	253 (42.7)		1413 (37.3)	72 (37.7)		2164 (47.9)	181 (45.1)	
I rarely or never drink milk	2629 (31.6)	206 (34.8)		1180 (31.2)	65 (34.0)		1449 (32.1)	141 (35.2)	
Missing	17 (0.2)	3 (0.5)		7 (0.2)	1 (0.5)		10 (0.2)	2 (0.5)	
Of milk, sour milk, and yoghurt, I drink/eat (Select only one option)			0.345			0.667			0.445

Mostly standard milk products (3% fat)	2684 (32.3)	180 (30.4)		1380 (36.5)	67 (35.1)		1304 (28.8)	113 (28.2)	
Mostly semi-skimmed products (1.5% fat)	3418 (41.1)	240 (40.5)		1500 (39.6)	77 (40.3)		1918 (42.4)	163 (40.6)	
Mostly low-fat products (0.5% fat)	1278 (15.4)	92 (15.5)		450 (11.9)	19 (9.9)		828 (18.3)	73 (18.2)	
I don't drink/eat dairy products	904 (10.9)	78 (13.2)		445 (11.8)	27 (14.1)		459 (10.2)	51 (12.7)	
Missing	23 (0.3)	2 (0.3)		11 (0.3)	1 (0.5)		12 (0.3)	1 (0.2)	
I eat porridge or muesli (Select only one option)			0.587			0.597			0.408
Rarely or never	4046 (48.7)	299 (50.5)		2042 (53.9)	108 (56.5)		2004 (44.3)	191 (47.6)	
A few times a week	2629 (31.6)	184 (31.1)		1010 (26.7)	51 (26.7)		1619 (35.8)	133 (33.2)	
Almost daily	1612 (19.4)	106 (17.9)		724 (19.1)	31 (16.2)		888 (19.6)	75 (18.7)	
Missing	20 (0.2)	3 (0.5)		10 (0.3)	1 (0.5)		10 (0.2)	2 (0.5)	
How often do you eat Keyhole*-labelled whole grain products such as coarse fibre-rich whole grain bread, oatmeal, muesli, or whole grain pasta? (Select only one option)			0.431			0.595			0.782
Three times a day or more often	191 (2.3)	19 (3.2)		85 (2.2)	7 (3.7)		106 (2.3)	12 (3.0)	
Twice a day	541 (6.5)	42 (7.1)		207 (5.5)	10 (5.2)		334 (7.4)	32 (8.0)	
Once a day	1968 (23.7)	145 (24.5)		767 (20.3)	39 (20.4)		1201 (26.6)	106 (26.4)	
A few times a week or less often	5567 (67.0)	384 (64.9)		2710 (71.6)	134 (70.2)		2857 (63.2)	250 (62.3)	
Missing	40 (0.5)	2 (0.3)		17 (0.4)	1 (0.5)		23 (0.5)	1 (0.2)	
I eat red meat (beef, pork, game, and lamb) or charcuterie products (ham, sausage, bacon) as a main meal (Select only one option)			0.287			0.106			0.217
Almost daily	1257 (15.1)	79 (13.3)		701 (18.5)	26 (13.6)		556 (12.3)	53 (13.2)	
A few times a week	4348 (52.3)	310 (52.4)		2032 (53.7)	118 (61.8)		2316 (51.2)	192 (47.9)	
Once a week	1432 (17.2)	96 (16.2)		616 (16.3)	30 (15.7)		816 (18.0)	66 (16.5)	
Rarely or never	1252 (15.1)	104 (17.6)		431 (11.4)	16 (8.4)		821 (18.2)	88 (21.9)	
Missing	18 (0.2)	3 (0.5)		6 (0.2)	1 (0.5)		12 (0.3)	2 (0.5)	

I eat whipped cream or crème fraîche with 25-40% fat (including in sauces or similar) (Select only one option)			0.753			0.758			0.856
A couple of times a week or more	2069 (24.9)	145 (24.5)		969 (25.6)	50 (26.2)		1100 (24.3)	95 (23.7)	
About once a week	3315 (39.9)	245 (41.4)		1518 (40.1)	80 (41.9)		1797 (39.7)	165 (41.1)	
Rarely or never	2906 (35.0)	200 (33.8)		1291 (34.1)	60 (31.4)		1615 (35.7)	140 (34.9)	
Missing	17 (0.2)	2 (0.3)		8 (0.2)	1 (0.5)		9 (0.2)	1 (0.2)	
I eat chips/crisps or cheese puffs			0.206			0.143			0.809
Almost daily	45 (0.5)	2 (0.3)		27 (0.7)	0 (0.0)		18 (0.4)	2 (0.5)	
A few times a week	697 (8.4)	36 (6.1)		389 (10.3)	11 (5.8)		308 (6.8)	25 (6.2)	
Once a week	3449 (41.5)	245 (41.4)		1621 (42.8)	89 (46.6)		1828 (40.4)	156 (38.9)	
Rarely or never	4103 (49.4)	307 (51.9)		1744 (46.1)	90 (47.1)		2359 (52.2)	217 (54.1)	
Missing	13 (0.2)	2 (0.3)		5 (0.1)	1 (0.5)		8 (0.2)	1 (0.2)	
I eat chocolate (Select only one option)			**0.028**			0.984			**0.011**
Almost daily	413 (5.0)	32 (5.4)		139 (3.7)	7 (3.7)		274 (6.1)	25 (6.2)	
A few times a week	2177 (26.2)	185 (31.2)		914 (24.1)	46 (24.1)		1263 (27.9)	139 (34.7)	
Once a week	3390 (40.8)	212 (35.8)		1572 (41.5)	81 (42.4)		1818 (40.2)	131 (32.7)	
Rarely or never	2316 (27.9)	160 (27.0)		1157 (30.6)	56 (29.3)		1159 (25.6)	104 (25.9)	
Missing	11 (0.1)	3 (0.5)		4 (0.1)	1 (0.5)		7 (0.2)	2 (0.5)	
I eat pastries (buns, Danish pastries, cookies, or cake) or ice cream (Select only one option)			0.110			0.683			0.234
Almost daily	170 (2.0)	8 (1.4)		86 (2.3)	3 (1.6)		84 (1.9)	5 (1.2)	
A few times a week	1548 (18.6)	117 (19.8)		709 (18.7)	39 (20.4)		839 (18.6)	78 (19.5)	
Once a week	3436 (41.4)	220 (37.2)		1553 (41.0)	71 (37.2)		1883 (41.7)	149 (37.2)	
Rarely or never	3135 (37.7)	245 (41.4)		1430 (37.8)	77 (40.3)		1705 (37.7)	168 (41.9)	
Missing	18 (0.2)	2 (0.3)		8 (0.2)	1 (0.5)		10 (0.2)	1 (0.2)	
I eat fruit and/or berries (fresh or frozen) (Select only one option)			0.904			0.581			0.803
Once a week or less often	1552 (18.7)	103 (17.4)		975 (25.8)	49 (25.7)		577 (12.8)	54 (13.5)	
A few times a week	3043 (36.6)	225 (38.0)		1513 (40.0)	81 (42.4)		1530 (33.8)	144 (35.9)	

Once a day	2290 (27.6)	164 (27.7)		893 (23.6)	47 (24.6)		1397 (30.9)	117 (29.2)	
Twice a day	1155 (13.9)	78 (13.2)		335 (8.8)	12 (6.3)		820 (18.1)	66 (16.5)	
At least three times a day	253 (3.0)	19 (3.2)		65 (1.7)	1 (0.5)		188 (4.2)	18 (4.5)	
Missing	14 (0.2)	3 (0.5)		5 (0.1)	1 (0.5)		9 (0.2)	2 (0.5)	
I eat a portion, equivalent to a handful, of various vegetables and/or root vegetables (fresh or frozen) (Select only one option)			0.448			0.677			0.205
Once a week or less often	633 (7.6)	48 (8.1)		445 (11.8)	22 (11.5)		188 (4.2)	26 (6.5)	
A few times a week	2485 (29.9)	163 (27.5)		1453 (38.4)	68 (35.6)		1032 (22.8)	95 (23.7)	
Once a day	3018 (36.3)	206 (34.8)		1310 (34.6)	68 (35.6)		1708 (37.8)	138 (34.4)	
Twice a day	1917 (23.1)	154 (26.0)		512 (13.5)	31 (16.2)		1405 (31.1)	123 (30.7)	
At least three times a day	237 (2.9)	18 (3.0)		58 (1.5)	1 (0.5)		179 (4.0)	17 (4.2)	
Missing	17 (0.2)	3 (0.5)		8 (0.2)	1 (0.5)		9 (0.2)	2 (0.5)	
I eat brown rice, whole grain pasta, or potatoes (Select only one option)			0.502			0.820			0.399
A few times a week or less often	6147 (74.0)	431 (72.8)		2877 (76.0)	144 (75.4)		3270 (72.3)	287 (71.6)	
Once daily	1871 (22.5)	134 (22.6)		813 (21.5)	43 (22.5)		1058 (23.4)	91 (22.7)	
More than once daily	265 (3.2)	24 (4.1)		90 (2.4)	3 (1.6)		175 (3.9)	21 (5.2)	
Missing	24 (0.3)	3 (0.5)		6 (0.2)	1 (0.5)		18 (0.4)	2 (0.5)	
I eat French fries or fried potatoes (Select only one option)			0.284			0.488			0.453
A few times a week	724 (8.7)	56 (9.5)		459 (12.1)	27 (14.1)		265 (5.9)	29 (7.2)	
Once a week	1963 (23.6)	128 (21.6)		1116 (29.5)	48 (25.1)		847 (18.7)	80 (20.0)	
A couple of times a month	3222 (38.8)	217 (36.7)		1395 (36.8)	69 (36.1)		1827 (40.4)	148 (36.9)	
Rarely or never	2380 (28.7)	188 (31.8)		809 (21.4)	46 (24.1)		1571 (34.7)	142 (35.4)	
Missing	18 (0.2)	3 (0.5)		7 (0.2)	1 (0.5)		11 (0.2)	2 (0.5)	
I eat fish or seafood (including as a sandwich topping) (Select only one option)			0.211			0.345			0.070
Once a month or less often	1461 (17.6)	112 (18.9)		761 (20.1)	35 (18.3)		700 (15.5)	77 (19.2)	
A couple of times a month	2008 (24.2)	129 (21.8)		940 (24.8)	40 (20.9)		1068 (23.6)	89 (22.2)	
Once a week	3003 (36.2)	224 (37.8)		1375 (36.3)	72 (37.7)		1628 (36.0)	152 (37.9)	

Twice a week	1435 (17.3)	107 (18.1)		568 (15.0)	38 (19.9)		867 (19.2)	69 (17.2)	
At least three times a week	374 (4.5)	17 (2.9)		132 (3.5)	5 (2.6)		242 (5.4)	12 (3.0)	
Missing	26 (0.3)	3 (0.5)		10 (0.3)	1 (0.5)		16 (0.4)	2 (0.5)	
I usually cut off visible fat when I eat (Select only one option)			**0.007**			0.947			0.164
Yes	5641 (67.9)	431 (72.8)		2056 (54.3)	104 (54.5)		3585 (79.3)	327 (81.5)	
No	2633 (31.7)	155 (26.2)		1717 (45.4)	86 (45.0)		916 (20.3)	69 (17.2)	
Missing	33 (0.4)	6 (1.0)		13 (0.3)	1 (0.5)		20 (0.4)	5 (1.2)	
How often do you eat candy, chocolate, sugar-sweetened soda, sugar-sweetened juice, or juice? (Select only one option)			0.601			0.918			0.416
2 times/day or more often	235 (2.8)	22 (3.7)		125 (3.3)	7 (3.7)		110 (2.4)	15 (3.7)	
1 time per day	833 (10.0)	56 (9.5)		405 (10.7)	21 (11.0)		428 (9.5)	35 (8.7)	
A few times a week	3360 (40.4)	241 (40.7)		1558 (41.2)	81 (42.4)		1802 (39.9)	160 (39.9)	
At most 1 time/week	3849 (46.3)	267 (45.1)		1687 (44.6)	81 (42.4)		2162 (47.8)	186 (46.4)	
Missing	30 (0.4)	6 (1.0)		11 (0.3)	1 (0.5)		19 (0.4)	5 (1.2)	
How often do you eat cake, cookies, biscuits, or other pastries? (Select only one option)			0.365			0.541			0.349
2 times/day or more often	32 (0.4)	4 (0.7)		20 (0.5)	2 (1.0)		12 (0.3)	2 (0.5)	
1 time per day	243 (2.9)	18 (3.0)		106 (2.8)	6 (3.1)		137 (3.0)	12 (3.0)	
A few times a week	2030 (24.4)	157 (26.5)		977 (25.8)	52 (27.2)		1053 (23.3)	105 (26.2)	
At most 1 time/week	5966 (71.8)	409 (69.1)		2671 (70.5)	130 (68.1)		3295 (72.9)	279 (69.6)	
Missing	36 (0.4)	4 (0.7)		12 (0.3)	1 (0.5)		24 (0.5)	3 (0.7)	

* Information about the Keyhole labelling system can be found at the Swedish Food Agency: https://www.livsmedelsverket.se/en/food-habits-health-and-environment/nyckelhalet

** “Bregott” is a mix of butter and rapeseed oil
